# Correlates of Sexual Minority Stress Profiles in Sexual Minority Men Living with HIV Who Use Stimulants

**DOI:** 10.1007/s10508-025-03319-8

**Published:** 2026-03-09

**Authors:** Renessa S. Williams, Ariana Johnson, Nicholas Metheny, Adam W. Carrico, Annesa Flentje

**Affiliations:** 1 School of Nursing and Health Studies, University of Miami, 5030 Brunson Dr Coral Gables, Coral Gables, FL 33146 USA; 2 Department of Public Health Sciences, University of Miami, Miami, FL USA; 3 College of Public Health and Social Work, Florida International University, Miami, FL USA; 4 School of Nursing Community Health Systems, University of California, San Francisco, San Francisco, CA USA

**Keywords:** Latent profile analysis, Sexual minority stress, Stimulant use, HIV, Sexual orientation

## Abstract

Sexually minoritized men (SMM) experience sexual minority stress, a product of exposure to prejudice, discrimination, and heterosexism. This study sought to identify the latent profiles of sexual minority stress among SMM with HIV who use methamphetamine and to examine the correlates associated with profile membership. A cross-sectional examination of 104 participants in San Francisco self-reported their experiences with sexual minority stress. A latent profile analysis was used to characterize the underlying “profiles” or subgroups of participants. ANOVA and Chi-square analysis were used to examine sociodemographic characteristics, psychological health, substance use, and HIV-related health associated with each profile. Participants’ mean age was 43 years old (SD = 9); most identified as Black, Hispanic, or Other racial and ethnic identities (57%), and had been diagnosed with HIV for 13 years (SD = 9) on average. Our findings revealed four distinct profiles: (1) High Concealment, Moderate Prejudice Events, (2) Low Concealment, High Prejudice Events, (3) Low Sexual Minority Stress, and (4) High Sexual Minority Stress. Members of the High Sexual Minority Stress profile had the highest mean addiction severity scores and the greatest endorsement of feelings of shame and guilt. Notably, each profile had clinically significant PTSD and depression scores, even among those who experienced stressful events to a lesser extent. Our findings suggest that sexual minority stress plays a critical role in substance use and mental health outcomes. Additional research is needed to elucidate the other facets of stress in SMM that use substances.

**Supplementary Information:**

The online version contains supplementary material available at 10.1007/s10508-025-03319-8.

## Introduction

Sexually minoritized men (SMM), including gay, bisexual, and other men who have sex with men, account for 70% of all new HIV infections in the USA (Centers for Disease Control & Prevention, 2023). While the incidence of HIV in this population is decreasing overall, progress has stalled—and even reversed—for men who exist at the intersection of multiple marginalized identities (Centers for Disease Control & Prevention, 2023). The minority stress model remains the prevailing theoretical explanation for HIV-related health inequities, in which sexual minority stress directly challenges physical health, mental health, and HIV symptom severity in SMM (Frost et al., 2015; Hatzenbuehler et al., 2008a; Meyer, 2003; Stephenson & Finneran, 2017). Sexual minority stress may be further described as distal stress, or instances of discrimination and prejudice, due to sexual minority status (Meyer, 2003). It can also take the form of proximal stress, an internal process that occurs as a result of distal stress, which may result in internalized heterosexism or concealment of sexual identity (Meyer, 2003).

Previous research suggests that distal and proximal stressors are both independently and interdependently associated with health outcomes (Flentje et al., 2020; Ramirez & Paz Galupo, 2019). Proximal stress processes have been more strongly predictive of biological outcomes, such as accelerated progression of HIV, including faster progression to AIDS and AIDS-related mortality (Flentje et al., 2020; Izard et al., 1993). In contrast, distal stressors are more strongly correlated with adverse mental health outcomes such as depression, anxiety, and post-traumatic stress disorder (PTSD). To this end, understanding both distal and proximal minority stress may be essential for capturing the complexity of minority experiences, predicting health outcomes, and guiding effective interventions and policies. Neglecting either dimension risks overlooking critical mechanisms driving health disparities.

Existing interventions designed to buffer the mental and physical effects of sexual minority stress have encompassed a combination of psychosocial (cognitive-behavioral therapy), behavioral (i.e., mindfulness) and interpersonal (i.e., social support/affirming relationships) strategies (Chaudoir et al., 2017). However, these interventions are often generalized to relatively homogenous samples of young, White gay men, despite evidence that minority stress is heightened in other communities (Chaudoir et al., 2017). SMM who exist at the intersection of multiple minoritized identities face even greater health inequities and marginalization as it relates to race (English et al., 2018; Everett et al., 2019; Storholm et al., 2019), ethnicity (Holloway et al., 2015), immigration status (Shramko et al., 2018), poverty (Storholm et al., 2019), housing instability (Flentje et al., 2016), and HIV-positive serostatus (Flentje et al., 2020). Developing interventions that effectively address how distal and proximal stress processes contribute to health outcomes in underrepresented groups may require tailored approaches. Without awareness of the proximal–distal minority stress distinction, clinicians risk misattributing symptoms, such as low mood, to a personality or anxiety disorder rather than recognizing them as adaptive responses to chronic stress (Nakamura et al., 2022; Pachankis et al., 2015). By integrating this understanding, providers can deliver more accurate, culturally responsive, and affirming care.

Exploration of minority stress profiles within a population at high risk for minority stress, suboptimal HIV care outcomes, and suboptimal mental health outcomes is warranted. SMM who use stimulants, including methamphetamine, powder cocaine, crack cocaine, and ecstasy, are especially vulnerable to HIV acquisition and face a great degree of marginalization (Carrico et al., 2008; Chahine et al., 2021; Ross et al., 2023). The United Nations estimates 30% of SMM who use stimulants are currently living with HIV in the USA (United Nations Office on Drugs and Crime, 2017). Stimulant use impairs users’ judgment and lowers risk aversion, which commonly increases sexual arousal and the desire to engage in sexual behavior (United Nations Office on Drugs and Crime, 2017). Combined, this can increase the likelihood of engaging in condomless sex, increase the frequency and duration of sex, and is shown to increase the number of sexual partners (Halkitis et al., 2014; Shoptaw & Reback, 2007; Woolf & Maisto, 2009), further increasing the risk of seroconversion. SMM with HIV who use stimulants have greater difficulties navigating the HIV care continuum, often have elevated viral loads, amplified HIV transmission risk, and faster HIV disease progression (Herek, 2009; McLellan et al., 1992; Mutumba et al., 2021). A growing body of literature suggests that sexual minority stress contributes to HIV disease progression in SMM with HIV who use stimulants (Flentje et al., 2020). One seminal study found that greater endorsement of internalized heterosexism was independently associated with fewer naive CD4 + and CD8 + T cells in a sample of SMM with HIV who used stimulants (Ghanooni et al., 2022). Studies further suggest sexual minority stress directly contributes to increased stimulant use and sexual risk behaviors (Hatzenbuehler et al., 2008b; Storholm et al., 2019). This may be partly explained by the depletion of psychosocial resilience, which has been shown to mediate the relationship between stress and stimulant use behaviors (Storholm et al., 2019). Further research is needed to elucidate how proximal and distal stressors affect this vulnerable population.

Studies have traditionally assessed sexual minority stress using additive, variable-centered approaches that inherently assume that all individuals experience sexual minority stress in the same way (Turan et al., 2019). Since individuals often do not know the specific stressors that contribute to their biological, psychological, and social well-being, traditional analytical approaches obfuscate important lessons on the linkage between minority stress and poor health outcomes. Several studies have suggested that modeling latent variables overcomes the challenges of capturing the complexity of discrimination experiences in diverse samples (Achterhof et al., 2019; Jenull & Wiedermann, 2015; Shramko et al., 2018). Specifically, latent profile analysis (LPA) uses continuous indicators to predict the probability of an individual’s membership within an inferred group from a given set of theoretically derived variables (Collins & Lanza, 2013; Spurk et al., 2020), allowing researchers to identify subgroups of individuals that share similar patterns or profiles. LPA has been described as a “person-centered” approach since participants themselves are clustered, rather than the variables that represent their individual attributes (Woo et al., 2018). A recent study employed an LPA approach to examine profile memberships significantly linked to the intensity of psychological distress, a documented mechanism of depression and negative coping responses (Frost et al., 2015; Frost & Meyer, 2009; Pennebaker, 1995) among sexually minoritized young adults (Shrader et al., 2024). Individuals in the moderate and high minority stress groups showed a higher likelihood of experiencing moderate and severe psychological distress than those in the low minority stress group (Shrader et al., 2024). Additionally, the group that concealed their sexual identity demonstrated an increased risk of severe psychological distress compared to the low minority stress group (Shrader et al., 2024). While these findings have been informative in describing the distal and proximal stress young adults experience, we have yet to understand the latent experiences of adult cohorts who may now have fewer overt stressors, but their proximal stress may be shaped by decades of living in less accepting contexts (Emlet et al., 2017).

In this study, we will examine sexual minority stress in SMM with HIV who use stimulants to provide a more nuanced understanding of sexual minority stress processes and their relationship to health outcomes using person-centered analytic methods. We hypothesized that (1) multiple latent classes would emerge from the data and (2) SMM in profiles with the least exposure to sexual minority stress would have the most optimal health outcomes (e.g., fewer mental health symptoms, less substance use behaviors, and undetectable viral loads) compared to SMM in profiles with greater sexual minority stress. To accomplish this, we used an LPA approach to identify subgroups of men who shared similar experiences of sexual minority stress. We then assessed whether subgroups defined by their distinct sexual minority stress experiences varied by sociodemographic characteristics, psychological health, substance use, and HIV-related care and treatment indicators.

## Method

### Participants

The data used in this study were collected from baseline survey responses to a positive affect intervention delivered during contingency management for stimulant abstinence in 184 SMM living with HIV in 2013–2017. Respondents were recruited from substance use disorder treatment programs, HIV medical clinics, AIDS service organizations, and referrals in Northern California. Eligible participants met the following inclusion criteria: (1) 18 years of age or older; (2) identify as a man with male sex assigned to them at birth; (3) reported anal sex with a man in the past 12 months; (4) documentation of HIV-positive serostatus (i.e., letter of diagnosis or ART medications matched to photo identification); and (5) provided a urine and/or hair sample that was reactive to methamphetamine use. The current cross-sectional study examined de-identified baseline surveys and peripheral venous blood samples from 104 participants. All participants provided signed informed consent.

### Measures

#### Sociodemographic Characteristics

Participants self-reported their age, gender (male, female, transgender), and race/ethnicity. Race/ethnicity was assessed as Black/African American not of Hispanic origin, Hispanic/Latino, Black Hispanic/Latino, White not of Hispanic origin, Asian/Pacific Islander, American Indian/Alaskan, Multiracial/Multi-cultural, or Other. In this analysis, responses were categorized as White non-Hispanic, Black non-Hispanic, Latino, and the remaining self-identifiers were condensed to the Other category. Sexual orientation was also examined using the Kinsey Scale, in which participants rated the degree to which they were exclusively heterosexual/straight or homosexual/gay on a seven-point Likert-type scale (Kinsey et al., 2003). Due to the small sample size, the response options were dichotomized as exclusively homosexual/gay, and all other responses were considered nonexclusively homosexual/gay. Moreover, the highest level of education completed (from less than 8th grade to completed graduate degree), employment status, and income were used to determine the socioeconomic characteristics. Lastly, unstable housing was assessed by asking participants if they were homeless or lived in a shelter in the past year, with response options of yes or no.

#### Sexual Minority Stress

Consistent with the minority stress model, stress was characterized by proximal and distal distinctions. Proximal stressors were represented by feelings of rejection, concealment of sexual identity, and internalized heterosexism. Distal stressors were interpreted as prejudice events, broadly defined as anti-gay violence and discrimination, as in previous studies (Meyer, 2003). Further descriptions of each measure are described below.

*Proximal Stress* Generalized sexual minority stress was measured using a validated five-item subscale derived from the Cultural Assessment of Risk for Suicide Sexual Minority Stress Scale (Chu et al., 2013). Statements reflected concealment (i.e., comfortability revealing sexual attractions and hiding sexual orientation to avoid distress), internalized stigma (i.e., self-directed negative beliefs about sexual orientation), and rejection from others (see Appendix). Each item was rated on a six-point Likert-type scale ranging from “strongly disagree” to “strongly agree” and demonstrated adequate internal consistency in our sample (Cronbach’s alpha = 0.81). Higher scores indicated higher levels of sexual minority stress.

The revised Internalized Homophobia Scale encompasses five items that assess one’s personal acceptance of their sexuality in the context of their value system and identity using a five-point Likert scale, ranging from “disagree strongly” to “agree strongly” (Herek, 2009). These items reflected acceptable reliability in our sample (Cronbach’s alpha = 0.85). Higher scores indicated higher internalized heterosexism. The Outness Inventory measured the extent to which participants disclosed their sexual minority status to friends, family, and the world (Mohr & Fassinger, 2000). Response options included a Likert-type scale that ranged from one (person definitely does not know about your sexual orientation status) to seven (person definitely knows about your sexual orientation status and it is openly talked about). In this study, outness was used as a proxy measure of concealment and showed good internal consistency (Cronbach’s alpha = 0.91). Items were reverse-scored to demonstrate that higher scores indicated a higher degree of concealing sexual identity.

*Distal Stress* Prejudice events were measured by six questions that assessed lifetime experiences of discrimination, such as being a victim of violence, threatened with physical violence, attended a church/religious setting with negative beliefs, or called names/insulted because of their sexual orientation (Cronbach’s alpha = 0.72). Binary response variables were standardized on a continuous scale, in which higher scores signified participants who had more frequent exposure to prejudice events.

#### Substance Use Factors

The Addiction Severity Index was administered, in which participants ascertained the number of days substances were used during the past month, their perceived impairment related to substance use, and their perceived need for substance use treatment (McLellan et al., 1992). Furthermore, an adapted five-item version of the Penn Alcohol Craving Scale was used to assess the frequency, intensity, and duration of cravings for methamphetamine, showing good internal consistency in our analysis (Cronbach’s alpha = 0.90) (Flannery et al., 1999; Mutumba et al., 2021). Higher scores indicated a higher methamphetamine craving.

#### Psychological Factors

Mental health was assessed using the Center for Epidemiological Studies–Depression (CES-D) scale, a 20-item measure that indicates the severity of symptoms associated with depression in the past week. The CES-D was scored continuously, with a higher value indicating greater symptoms of depression (Lewinsohn et al., 1997) (Cronbach’s alpha = 0.90). Scores averaged at 16 or above were considered at risk of clinical depression. Additionally, the Post-Traumatic Stress Disorder Checklist–Civilian is a self-report rating scale comprised of 17 items that correspond to symptoms of PTSD (Bliese et al., 2008). Scores were examined continuously, and higher scores suggested that the participant had more frequent exposure to trauma (Cronbach’s alpha = 0.92). Scores of 30 or above on average were considered to have a moderate-to-high risk of a PTSD diagnosis (Bliese et al., 2008).

The Coping Self-Efficacy Scale is a 26-item measure administered to assess the degree to which one may cope with various life challenges (Chesney et al., 2006). It consists of three subscales that measure the use of problem-focused coping (i.e., efforts to control or manage the source of stress), emotion-focused coping (i.e., efforts to regulate emotional responses to sources of stress that are caused by uncontrollable events), and receiving social support to cope with life challenges. Internal consistency is acceptable across all subscales (Cronbach’s alpha = 0.85, 0.84, and 0.77, respectively). The mean scores are assessed for each subscale, with higher scores indicating a greater ability to cope.

The Differential Emotions Scale is a widely used 36-item instrument in which participants rate their feelings toward fundamental emotions over the past week (Izard & Read, 1982; Izard et al., 1993). The scale is comprised of emotions that capture positive affect (enjoyment, excitement, interest, joy, and surprise), shame (negative self-evaluation) (Batchelder et al., 2022), and guilt (negative evaluation of behaviors) (Batchelder et al., 2022; Dearing et al., 2005) on a five-point Likert scale (never–very often). Composite scores for each subscale are assessed separately. Higher mean scores represent higher levels of positive affect, shame, and guilt (Cronbach’s alpha = 0.87, 0.76, and 0.83, respectively).

#### HIV-Related Care and Treatment Indicators

Participants reported the number of years they had been diagnosed with HIV and the length of time since ART was first initiated. Engagement in HIV-related care was measured by the sum of four binary questions, with higher scores indicating greater care engagement, as done in previous studies (Carrico et al., 2016; Jin et al., 2018). The questions included the following: (1) Were you taking any HIV medications?; (2) Did you schedule any medical appointments with an HIV primary care provider?; (3) Was your most recent HIV viral load test undetectable?; and (4) Did you have blood drawn to measure your CD4 + T cell count or HIV viral load? Lastly, CD4 + T cell count was measured with whole blood using flow cytometry in a commercial laboratory.

### Statistical Analysis

To test Hypothesis 1, we used an LPA approach to model subgroups that shared homogenous experiences of sexual minority stress. Underlying patterns of covariance in the data structure revealed “profiles” or subgroups of participants based on their experiences with distal and proximal stress indicators: (1) generalized sexual minority stress, (2) internalized heterosexism, (3) outness, and (4) prejudice events. Each survey item was treated as a continuous variable from 1 (low sexual minority stress) to 10 (high sexual minority stress) using z-standardized mean scale scores, a method that has been previously described (Spurk et al., 2020). We calculated the item response means and overall sample means for each subscale to make comparisons across profiles.

The most parsimonious model was chosen by iteratively comparing several model fit indices for two-to-five-profile solutions. First, we examined the Bayesian Information Criteria (BIC), adjusted Bayesian Information Criteria (ABIC), Lo–Mendell–Rubin-adjusted likelihood ratio test (LMR), and parametric bootstrap likelihood ratio test (BLRT). BIC and ABIC are interpreted such that the lowest value is considered the best fit (Achterhof et al., 2019). The LMR and BLRT both provide comparisons between models, such that significant values indicate that the model has an improved fit over the model with fewer profiles. Second, entropy was assessed to determine the probability of each participant’s likelihood to belong to each profile. Lower entropy values (<.9) may indicate that the model does not represent profiles with uniquely separate characteristics. Finally, theoretical coherence was considered to evaluate the interpretability of the findings.

#### Correlates of Sexual Minority Profiles

We calculated the descriptive means, standard deviations, and ranges for each profile based on participants’ sociodemographic characteristics, psychological factors, substance use behaviors, and HIV-related health. To test Hypothesis 2, we conducted ANOVA tests for continuous variables, and chi-square tests for categorical variables were used to examine differences across each profile. Significant variables identified via omnibus tests were followed by Tukey’s test for pairwise mean comparisons to further evaluate groups with the greatest differences. *p*-values were considered significant at 0.05. Statistical analyses were performed using the MPlus and R software (Muthen & Muthen, n.d.; R Core Team, 2022). The materials and analysis codes used in this study are available upon request.

## Results

### Description of the Sample

Of the 184 recruited participants, 110 completed baseline interviews and peripheral venous blood draws. An additional six participants were excluded due to missing data. The final sample included 104 cisgender SMM. The majority of the sample identified as a racial/ethnic minority (56.7%): 16.3% Black, 26.9% Latino, and 13.5% Multiracial/Other, with the remaining 43.3% identified as non-Hispanic White. The average age of the participants was 43.7 years old (SD = 8.98), predominantly identified as exclusively homosexual or gay (73%), and 46.2% reported using injection drugs in the past 30 days. Individuals had lived with HIV for 13 years on average, had been prescribed ART for approximately 11 years (SD = 1.42), and CD4 + T cell counts were averaged in the normal range (M = 663 ml/copy; SD = 44.34). Notably, the average PTSD (46.49, SD = 14.30) and depression (24.8, SD = 11.4) scores were clinically significant. Table 1 further describes the mean scores and frequencies of each variable by profile.

**Table 1:** Sociodemographic characteristics, psychological factors, substance use behaviors, and HIV-related care and treatment indicators by Profile Membership (*n* = 104)

Continuous characteristics	Profile 1: *n* = 23	Profile 2: *n* = 37	Profile 3: *n* = 28	Profile 4: *n* = 16	
	Mean (SD)	Mean (SD)	Mean (SD)	Mean (SD)	
Age**	45.9 (7.37)	40.9 (9.72)	41.3 (9.09)	46.5 (6.52)	***F* = 2.84; *p* =.04**
Addiction Severity**	0.11 (0.09)	0.16 (0.09)	0.15 (0.07)	0.24 (0.13)	***F* = 5.78; *p* =.0011**
Depression**	19.0 (9.18)	26.3 (12.5)	23.4 (10.1)	31.9 (9.74)	***F* = 4.92; *p* =.003**
PTSD**	42.4 (12.3)	48.5 (15.8)	43.4 (13.1)	59.1 (10.5)	***F* = 5.79; *p* =.001**
Shame**	2.19 (0.89)	2.66 (0.95)	2.45 (0.96)	3.21 (0.74)	***F* = 4.22; *p* =.007**
Guilt**	2.32 (0.98)	2.90 (1.03)	2.74 (0.97)	3.65 (0.79)	***F* = 6.03; *p* =.0008**
Time HIV	11.8 (7.5)	14.2 (9.9)	11.9 (7.68)	14.6 (7.5)	*F* = 0.75; *p* =.5251
Time ART	10.8 (7.7)	10.9 (7.3)	8.6 (5.9)	12.0 (6.6)	*F* = 0.85; *p* =.4720
Positive affect	33.2 (8.79)	33.5 (8.32)	31.2 (8.84)	30.4 (6.21)	*F* = 0.80; *p* =.4947
Employment status	0.79 (0.22)	0.82 (0.15)	0.74 (0.22)	0.78 (0.22)	*F* = 1.02; *p* =.3871
Engaged In Care	2.96 (1.36)	3.41 (0.92)	3.25 (1.17)	3.13 (1.31)	*F* = 0.75; *p* =.5244
Problem-focused Coping**	6.57 (1.72)	6.65 (2.03)	5.96 (2.17)	4.89 (2.29)	***F* = 3.15; *p* =.0283**
Emotion-focused Coping**	6.34 (1.71)	5.08 (2.23)	5.04 (2.41)	4.38 (1.96)	***F* = 3.04; *p* =.0326**
Social support	6.52 (2.04)	5.93 (2.57)	5.90 (2.70)	5.17 (2.07)	*F* = 0.98; *p* =.4051
Alcohol Cravings	2.52 (1.36)	3.19 (1.49)	2.66 (1.45)	3.49 (1.46)	*F* = 2.14; *p* =.0998
CD4 + T cell counts	623 (341.4)	659.3 (305.7)	644.9 (285.4)	725.9 (294.8)	*F* = 0.37; *p* =.7728
Categorical characteristics					
Race					(*P*) = <.01; *p* =.06
Black	5 (21.7%)	3 (8.1%)	6 (21.4%)	3 (18.8%)	
White	8 (34.8%)	20 (54.1%)	14 (50%)	3 (18.8%)	
Latino	6 (26.1%)	10 (27.0%)	6 (21.4%)	6 (37.4%)	
Other	4 (17.4%)	4 (10.8%)	2 (7.2%)	4 (25%)	
Education					(*P*) = <.001; *p* =.25
High school grad or less	21 (91.3%)	23 (62.2%)	20 (71.4%)	10 (62.5%)	
Some college/trade school	1 (4.35%)	10 (27.0%)	6 (21.4%)	4 (25.0%)	
College grad or higher	1 (4.35%)	4 (10.8%)	2 (7.2%)	2 (12.5%)	
Income					(*P*) = <.01; *p* =.71
Less than $50,000	18 (78.3%)	25 (67.6%)	17 (60.7%)	9 (56.3%)	
$50,000–$100,000	5 (34.8%)	8 (21.6%)	9 (32.1%)	5 (31.2%)	
$100,000 +	0 (0%)	4 (10.8%)	2 (7.1%)	2 (12.5%)	
Sexual Orientation					**(*P*) <.0001; *p* =.01**
Exclusively homosexual or gay	16 (69.6%)	33 (89.2%)	19 (67.9%)	8 (50%)	
Nonexclusively homosexual or gay	7 (30.4%)	4 (10.8%)	8 (28.5%)	8 (50%)	
Unstably housed in the past year**					**(*P*) =.002; *p* =.05**
Yes	4 (17.4%)	1 (2.7%)	1 (3.6%)	3 (18.8%)	
No	19 (82.6%)	36 (97.3%)	27 (96.4%)	13 (81.2%)	
Injection drugs use (30 days)					(*P*) =.044; *p* =.82
Yes	10 (43.5%)	17 (45.9%)	15 (53.6%)	9 (56.3%)	
No	13 (56.5%)	20 (54.1%)	13 (46.4%)	7 (43.7%)	

(*P*) = Fisher's exact; **p-value <.05

### Characteristics of Profiles

**Fig. 1:**
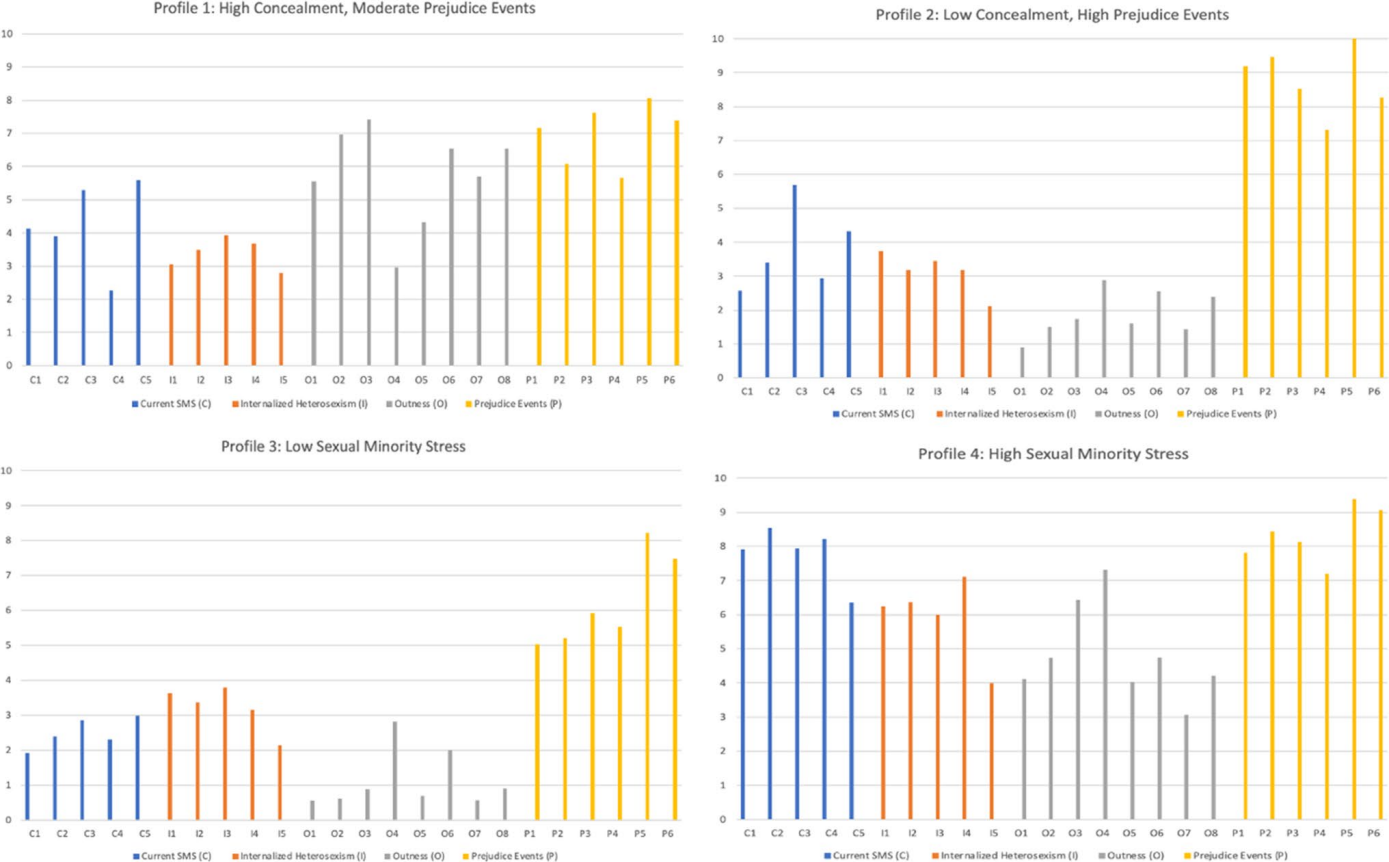
Mean scores of each sexual minority stress variable by profile

Table 2 presents the fit statistics for the two-to five-class solutions. In line with Hypothesis 1, a four-profile solution was identified as the best-fitting model because it had the lowest AIC, BIC, and ABIC while still maintaining uniquely distinct groups representing no less than 5% of the total sample size per profile (He & Fan, 2018). Figure 1 shows the frequency of endorsing each sexual minority stress indicator based on the four-profile model comprised of 23 individuals in Profile 1 (22%), 37 individuals in Profile 2 (36%), 28 individuals in Profile 3 (27%), and 16 individuals in Profile 4 (15%).

**Table 2:** Fit statistics of latent profile analysis (*n* = 104)

No. of Profiles	AIC	BIC	ABIC	Entropy	LMR LRT	BS LRT
2 Profile	8740.995	8949.902	8700.341	0.98	515.899; *p* =.01	520.013; *p* = <.01
3 Profile	8573.297	8853.602	8518.748	0.97	219.944; *p* =.41	221.698; *p* = <.01
4 Profile	**8485.499**	**8837.203**	**8417.055**	**0.984**	140.677; *p* =.73	**141.798; *p* = <.01**
5 Profile	8443.46	8866.563	8361.122	0.982	95.279; *p* =.76	96.038; *p* = <.01

Profile 1 had the highest mean outness score, indicating a high degree of respondents who concealed their sexual orientation. Profile 1 had the highest frequency of concealed sexual orientation with straight friends (55.4%), work peers (69.7%), work supervisors (74.2%), father (65.4%), mother (43.3%), siblings (56.9%), and extended family (65.5%). Mean scores associated with generalized sexual minority stress experiences and internalized stigma were lower than the overall sample means. Profile 1 members notably endorsed wishing that they were not gay/bisexual (30%) at the lowest frequency compared to the other profiles. Prejudice events were also reported at moderate frequencies; thus, this profile was referred to as High Concealment, Moderate Prejudice Events.

Profile 2 reflected respondents who scored well above the overall sample mean for the prejudice event subscale. Respondents reported the highest frequency of anti-gay violence (92%), threats of anti-gay violence (94.7%), discrimination (85.1%), and anticipated discrimination in the next year (73.1%), and approximately 100% have been called names or insulted in their lifetime due to sexual orientation. Internalized stigma scores were lower than the overall sample means. Members of Profile 2 were the least likely to endorse wanting to stop being gay/bisexual (31.8%) and wanting to accept an opportunity to change their sexual orientation if given a chance (34.5%) or stop being gay/bisexual through professional means (21.1%), compared to the other profiles. Furthermore, participants were more likely to disclose their sexual orientation, as evidenced by the lower frequency of concealment compared to the overall sample means. Accordingly, this profile was labeled Low Concealment, High Prejudice Events.

Members of Profile 3 were evaluated as having the lowest frequency of generalized sexual minority stress, concealing their sexual orientation, internalized heterosexism, and prejudice events compared with the other profiles; thus, this profile was interpreted as Low Sexual Minority Stress. This profile was used as the reference group for subsequent analysis. Conversely, Profile 4 had the highest mean frequency of generalized sexual minority stress and internalized stigma compared with the other profiles; therefore, membership is referred to as High Sexual Minority Stress. Of note, these members were most likely to endorse their sexual orientation to strangers (73.1%) rather than to family, friends, or coworkers. Members of this group also reported the highest frequency of attending a church that had negative beliefs about gay or lesbian people at least once in their lifetime (90.1%).

### Associations of Sociodemographic Characteristics Across Profiles

To further examine these differences, we conducted pairwise comparison tests, as shown in Table 3. Statistically significant differences were observed across these profiles based on sociodemographic characteristics, including age (*F* = 2.84; *p* =.04), sexual orientation (Fisher's exact (*p*) =  <.0001; *p* =.01), and reports of homelessness in the past year [(*p*) =.002; *p* =.05)]. Our pairwise findings show that these distinctions were driven by differences between membership in the High Sexual Minority Stress subgroup and the Low Concealment, High Prejudice Event subgroup. In general, participants in the High Sexual Minority Stress subgroup were the oldest (46.5 years old; SD = 6.52), had the highest mean reports of unstable housing in the past year (19%), and had the lowest proportion of exclusively homosexual/gay attractions (50%) compared with those classified in the Low Concealment, High Prejudice Event subgroup. On the other hand, participants in the Low Concealment, High Prejudice Event group were the youngest participants on average (40.9 years; SD = 9.72), had the lowest mean reports of unstable housing in the past year (3%), and had the highest proportion of exclusively homosexual/gay attractions (89%).

**Table 3:** Pairwise comparisons of variables significant in omnibus test

Variable	Comparison	Difference between means (95% confidence intervals)	*p*-value <.05
Addiction severity	Profile 4 versus Profile 1	0.13 (0.05, 0.21)	***
	Profile 4 versus Profile 3	0.09 (0.01, 0.17)	***
Depression	Profile 4 versus Profile 1	12.89 (3.72, 22.07)	***
PTSD	Profile 4 versus Profile 1	16.63 (5.03, 28.23)	***
	Profile 4 versus Profile 3	15.63 (4.47, 26.80)	***
Shame	Profile 4 versus Profile 1	1.02 (0.24, 1.80)	***
	Profile 4 versus Profile 3	0.76 (0.01, 1.50)	***
Guilt	Profile 4 versus Profile 1	1.33 (0.50, 2.15)	***
	Profile 4 versus Profile 3	0.91 (0.11, 1.70)	***
Problem-focused coping	Profile 2 versus Profile 4	1.76 (0.16, 3.36)	***
Emotion-focused coping	Profile 1 versus Profile 4	1.96 (0.14, 3.78)	***
Sexual orientation	Profile 2 versus Profile 4	0.39 (0.06, 0.73)	***
Age	Profile 4 versus Profile 2	2.38 (1.51,2.71)	***
	Profile 4 versus Profile 3	2.21 (1.15, 3.01)	***

Profile 1: High Concealment, Moderate Prejudice Events; Profile 2: Low Concealment, High Prejudice Events; Profile 3: Low Sexual Minority Stress; Profile 4: High Sexual Minority Stress

### Associations of Psychological Factors Across Profiles

Next, mental health was further examined by profile, noting significant differences in depression (*F* = 4.92; *p* =.003) and PTSD (*F* = 5.79; *p* =.001) severity. The High Sexual Minority Stress subgroup included participants who had the highest mean depression scores (M = 31.9; SD = 9.74) and PTSD scores (M = 59.1; SD = 10.5) compared to the High Concealment, Moderate Prejudice Event group (M = 19.0; SD = 9.18, M = 42.4 SD = 12.3, respectively), and the Low Sexual Minority Stress subgroup (M = 43.4; SD = 13.1).

Subsequently, coping and differential emotions were examined according to profile. Significant differences were observed for shame (*F* = 4.22, *p* =.007), guilt (*F* = 6.03, *p* =.0008), problem-focused coping (*F* = 3.15, *p* =.0283), and emotion-focused coping (*F* = 3.04, *p* =.0326). The High Sexual Minority Stress subgroup also had the greatest mean difference in shame scores (M = 3.21; SD = 0.74) compared to the High Concealment, Moderate Prejudice Event subgroup (M = 2.19; SD = 0.89), and the Low Sexual Minority Stress subgroup (M = 2.45; SD = 0.96). Similar differences were observed in the comparison of guilt scores between the High Sexual Minority Stress subgroup (M = 3.65; SD = 0.79) and the High Concealment, Moderate Prejudice Event subgroup (M = 2.32; SD = 0.98), as well as the Low Sexual Minority Stress subgroup (M = 2.74; SD = 0.97). Moreover, individuals in the Low Concealment, High Prejudice Event group had higher problem-focused coping mean scores (M = 6.65; SD = 2.03) than those in the High Sexual Minority Stress subgroup (M = 4.89; SD = 2.29). Likewise, the High Concealment and Moderate Prejudice Event groups had the highest mean emotion-focused coping scores (M = 6.34; SD = 1.71) compared to the High Sexual Minority Stress group (M = 4.38; SD = 1.96).

### Associations of Substance Use Factors Across Profiles

Only addiction severity differed significantly across profiles (*F* = 5.78; *p* =.0011). Individuals in the High Sexual Minority Stress subgroup had significantly higher mean addiction severity scores (M = 0.24; SD = 0.13) than those in the High Concealment, Moderate Prejudice Event subgroup (M = 0.11; SD = 0.09) and Low Sexual Minority Stress subgroup (M = 0.15; SD = 0.07).

### Associations of HIV-Related Health Indices Across Profiles

There were no statistically significant differences found by profile among the HIV-related health indicators. Most participants in the sample were ART-adherent and living with treated HIV.

## Discussion

The current study is one of the first to examine sexual minority stress typologies and their correlates according to latent profile membership among SMM with HIV who used stimulants. Four typologies were identified: High Concealment, Moderate Prejudice Events (Profile 1), Low Concealment, High Prejudice Events (Profile 2), Low Minority Stress (Profile 3), and High Minority Stress (Profile 4). The profiles differed significantly by age, sexual orientation, addiction severity, coping self-efficacy, depression, PTSD, shame, guilt, and housing status. Despite distinct experiences of sexual minority stress, each profile had clinically significant PTSD and depression risk scores. These findings suggest that sexual minority stress processes may play a significant role in depression and PTSD risk, even among those with relatively few exposures. Furthermore, this study contributes to the growing literature by recognizing the complex range of sexual minority stress processes and the need for strategies to reduce mental health burdens among a diverse sample of SMM with HIV who use stimulants.

We hypothesized that profiles with the lowest co-occurring proximal and distal stresses would significantly correlate with optimal health outcomes. Instead, we found that SMM with moderate exposure to prejudice events who highly concealed their sexual orientation (Profile 1) had the lowest mean depression and PTSD symptom severity. Although one previous LPA of sexual minority stress profiles showed that high concealment of sexual orientation contributes to psychological distress (Shrader et al., 2024), the minority stress model considers concealment as both harmful and protective (Meyer, 2003). SMM may find it advantageous to conceal their sexual orientation by presenting as “straight” to avoid stigma or systemic exclusion (Meyer, 2003). However, concealing one’s sexual orientation can also be a source of stress. The act of constantly monitoring actions to conform to heterosexist norms and maintain secrecy may require considerable effort. There are several possible explanations for this finding. First, SMM, who more frequently concealed their sexual orientation from their close networks, was mostly endorsed by racial and ethnically minoritized individuals. Considering the racial, ethnic, and gendered intersections of stress, in addition to the systemic embedding of racism, sexism, and heterosexism, it is plausible that individuals in this group developed resilience out of necessity and due to the constant exposure to stress that may be layered by the effects of these minoritized identities (Bowleg, 2008, 2012; Shramko et al., 2018). Evidence suggests that concealment is a protective mechanism in racial and ethnically minoritized SMM, whereas it is more harmful in White SMM (Meyer, 2003; Vincent et al., 2021). White SMM have been shown to have greater social support after disclosing their sexual minority status, whereas racial and ethnically minoritized SMM have reported less support and even more instances of discrimination (Vincent et al., 2021). We recognize the limitation in our study that assessing sexual minority stress alone presents a limited scope of the various stressors one may endure; however, our findings highlight the importance of considering an intersectional approach to gain a better understanding of the interdependent effects of minoritized identities and their interaction with structural barriers that can affect the health of SMM in future studies (Algarin et al., 2019; Bauer, 2014).

The highest frequency of shame, guilt, substance use, PTSD, and depression symptoms was observed in groups with the greatest instances of prejudice events, particularly among SMM characterized by concealing their sexual orientation to a higher degree (Profile 4) and SMM who endorsed concealing their sexual orientation to a lesser degree (Profile 2). Although we cannot determine causal relationships in this study, there are established linkages between substance use behaviors and psychological health outcomes (Batchelder et al., 2022; Chahine et al., 2021; Dearing et al., 2005). For instance, shame is a key contributor to negative self-perceptions, exacerbates drug-use behaviors, and is closely linked to depression (Batchelder et al., 2022). In light of the findings from this study, it is plausible that stimulants are used to cope with prejudice events, leading to shame that amplifies negative devaluations (Dearing et al., 2005). Overall, our findings suggest that prejudice events are key indicators of poor health outcomes.

Coping is proximally associated with stressful events and psychological vulnerabilities; however, the literature has not been representative of the unique context of sexual minority stress experienced in SMM with HIV who use stimulants (Chesney et al., 2006; Ouch & Moradi, 2019). From the previous research, we expected that groups that scored highly on emotion-focused coping, problem-focused coping, and social support would be strongly associated with the low minority stress group (Chesney et al., 2006; Ouch & Moradi, 2019). However, SMM who highly concealed their sexual orientation from others and experienced a moderate degree of prejudice events (Profile 1) had significantly greater emotion-focused and problem-focused coping skills and reported the lowest levels of depression and PTSD symptoms relative to individuals with high minority stress (Profile 4). Many factors have been linked to coping styles. For example, external acts of discrimination are often uncontrollable, fixed acts, which are perpetrated by others. As such, a recent meta-analysis suggests that utilizing problem-focused coping skills when faced with an uncontrollable event may not be sufficient enough to overcome its impact in adults (Penley et al., 2002). Different coping styles may be necessary (e.g., social support and/or emotion-focused coping) to overcome external acts of discrimination. A recent study of SMM who experienced discrimination found that problem-focused and emotion-focused coping strategies have been successful in reducing stress (Ouch & Moradi, 2019); however, more research is needed to understand optimal coping strategies that are effective against sexual minority stress. The current study provides initial evidence of the salience of emotion-focused coping and the need to further investigate other adaptive coping strategies and their role in sexual minority stress consequences (Ouch & Moradi, 2019; Sandfort et al., 2009).

Sociodemographic characteristics differed significantly across each latent subgroup. SMM in groups who highly concealed their sexual orientation were older, mostly racial or ethnically minoritized, and had a higher proportion of individuals who were unstably housed at least once in the past year. Our findings suggest great variability in the way sexual minority stress is experienced across generational cohorts. Older adults are frequently cited as having greater sexual minority stress than younger individuals and are often attributed to witnessing the historical revolutions in sociopolitical views of HIV and sexuality over the past 40 years (Emlet et al., 2015; McCormack et al., 2014; Williams et al., 2020). The differences in our sample may be related to the ever-changing norms and values attributed to HIV acquisition, masculinity, substance use, and gender norms. Older individuals may possess a more comprehensive grasp of the stigma associated with HIV, not only because they have amassed more stressful experiences over time, but also because of their awareness of the stigmatization faced by people diagnosed with HIV during the early stages of the epidemic. This understanding is rooted in the historical context when treatment was neither available nor accessible, and HIV-related mortality was prevalent. Moreover, given that the study findings are contextualized to the San Francisco Bay area, unstable housing disproportionately impacts SMM of color (Corliss et al., 2011; Wilson et al., 2020). Reasons for homelessness have been associated with proximal (Bruce et al., 2014; Kidd, 2007) as well as distal sexual minority stress (Flentje et al., 2016). Longitudinal examinations of sexual minority stress as a mediator of unstable housing and onset of mental and physical health symptoms are warranted.

Members of the high sexual minority stress subgroup (Profile 4) distinctly had a high frequency of endorsing generalized sexual minority stress, internalized heterosexism, and concealing sexual orientation. Our findings parallel a previous study that found experiencing both distal and proximal stress predicted greater mental health problems than either individual domain alone (Ramirez & Paz Galupo, 2019). Furthermore, approximately half of the individuals in this profile endorsed having both male and female sexual attractions/behaviors. These findings suggest stress is amplified when one is sexually and romantically attracted to multiple genders. In line with previous work, there is strong evidence that those exclusively attracted to multiple genders are at greater risk of mental and physical health problems compared to monosexual (i.e., straight or gay/lesbian) individuals (Feinstein & Dyar, 2017; Meyer, 2003). Research often does not distinguish between attractions and sexual behaviors in analytic samples (Meyer, 2003; Ramirez & Paz Galupo, 2019); however, this distinction is important because there exists an invisibility of individuals who are attracted to more than one gender or do not regard gender as binary (Meyer, 2010). Future research that separately considers behavior, attraction, and identity-driven sexuality may better represent the unique experiences of SMM.

Despite the unique strengths of our study, there are limitations that must be considered in light of the findings. First, it relied on cross-sectional data; therefore, causal assumptions could not be made. More longitudinal work is necessary to examine the health consequences of sexual minority stress by profile membership, as previous studies have suggested that proximal and distal stress are dynamic processes (Puckett et al., 2018). Next, due to the small sample size, we could not determine intra-categorical heterogeneity within Black, Hispanic, White, or Other racial and ethnic identities. This is an important area for future work, as SMM of racial and ethnically minoritized backgrounds are often underrepresented in research; thus, we lose important information on the intersectional nuances of minority stress (Flentje et al., 2020; Vincent et al., 2021). Furthermore, the study was conducted in the San Francisco Bay Area, a setting rich with resources and extensive services for SMM with HIV who use stimulants (Carrico et al., 2014). This may partially explain the lack of differences observed between sexual minority stress profiles and HIV-related care and treatment outcomes. Furthermore, our sample mostly consisted of ART-treated SMM in care, so our findings may not be generalizable to the larger US population. Further investigations are necessary to examine HIV-related care and treatment among those who experience sexual minority stress in resource-poor settings. Finally, this study did not include structural determinants and other potential confounding variables that may have affected the study findings, given that the potential burden of dealing with sexual minority stress should not be on individuals, but on the systems that perpetuate discrimination/stigma. Future studies could benefit from the inclusion and control of such variables to allow for a more nuanced interpretation of the results. Nevertheless, this study is an important step in understanding the confluence of proximal and distal sexual minority stress in a population with multiple marginalized identities and its association with psychological, HIV engagement, and substance-related outcomes.

Overall, the study’s findings have important clinical implications for interventions that seek to mitigate sexual minority stress and provide an initial understanding of sexual minority stress typologies. This study may be useful to help clinicians identify SMM at risk of depression, PTSD, and addiction, especially among those who use stimulants and are living with HIV. We propose that robust protective and preventative approaches are needed to support SMM, regardless of exposure to sexual minority stress. Future research that incorporates an intersectional lens may further disentangle the mental and physical health effects of minority stress.

## Supplementary Information

Below is the link to the electronic supplementary material.

Supplementary file1 (DOCX 16 KB)

## Data Availability

This study is not formally registered. Similarly, the analysis plan is not formally pre-registered. De-identified data, analytic codes, and materials are available from the authors upon reasonable request and all necessary institutional approval.

## Code Availability

All analyses were conducted using MPlus and R software (Muthen & Muthen, n.d.; R Core Team, 2022). Codes may be made available upon request.
