# Supplementary material for: Correlates of Sexual Minority Stress Profiles in Sexual Minority Men Living with HIV Who Use Stimulants
Source: Arch Sex Behav. 2026 Mar 9;55(2):911–23. doi: 10.1007/s10508-025-03319-8 (PMC13048913; doi:10.1007/s10508-025-03319-8)
Supplement: Supplementary file 1 — Supplementary file1 (DOCX 16 KB) [file 10508_2025_3319_MOESM1_ESM.docx]

**Appendix**

Proximal and Distal Sexual Minority Stress Items

| Generalized  Sexual Minority Stress | C1 | The decision to hide or reveal my sexual orientation to others causes me significant distress. |
| --- | --- | --- |
|  | C2 | Because of my sexual orientation, no one understands my pain or distress. |
|  | C3 | I was rejected by a family member or friend after telling him/her my sexual orientation. |
|  | C4 | I feel confused or conflicted by my sexual orientation. |
|  | C5 | I feel comfortable revealing my sexual attractions and/or behavior. |
|  | C6 | People treat me unfairly because of my sexual identity. |
|  | C7 | At times, I feel I stick out because of my sexual attractions. |
|  | C8 | Stereotypes about gay and bisexual people hurt my self-esteem or the way I see myself. |
| Internalized Heterosexism | I1 | I wish I weren’t gay/bisexual. |
|  | I2 | I have tried to stop being attracted to men in general. |
|  | I3 | If someone offered me the chance to be completely heterosexual, I would accept the chance. |
|  | I4 | I feel that being gay/bisexual is a personal shortcoming for me. |
|  | I5 | I would like to get professional help in order to change my sexual orientation from gay/bisexual to straight. |
| Outness | O1 | To which degree do **your new straight friends** know about your sexual orientation? |
|  | O2 | To which degree do **your** **work peers** know about your sexual orientation? |
|  | O3 | **Your work supervisors**? |
|  | O4 | **Strangers**? |
|  | O5 | **Your mother?** |
|  | O6 | **your** f**ather** |
|  | 07 | **Your siblings?** |
|  | 08 | **Your extended family relatives?** |
| Prejudice  Events | P1 | Have you **ever** been the victim of anti-gay violence? That is, were you harmed because you were gay? |
|  | P2 | Have you **ever** been threatened with physical violence as a result of your sexual orientation? That is were you threatened because you were gay? |
|  | P3 | Have you **ever** been discriminated against in any way because of your sexual orientation? |
|  | P4 | In the **next year**, do you believe you will experience discrimination, threats, or violence due to your sexual orientation? |
|  | P5 | Have you **ever** been called names or insulted because of your sexual orientation? |
|  | P6 | Have you **ever** attended a church that held negative beliefs about gay or lesbian people? |
